# Applications of Airway Ultrasound for Endotracheal Intubation in Pediatric Patients: A Systematic Review

**DOI:** 10.3390/jcm12041477

**Published:** 2023-02-13

**Authors:** Yijun Liu, Wei Ma, Jin Liu

**Affiliations:** 1 Department of Anaesthesiology, West China Hospital, Sichuan University, Chengdu 610041, China; 2 The Research Units of West China (2018RU012)-Chinese Academy of Medical Sciences, West China Hospital, Sichuan University, Chengdu 610041, China

**Keywords:** airway ultrasound, endotracheal intubation, pediatric, systematic review

## Abstract

Endotracheal intubation is a challenging procedure for pediatric patients. Airway ultrasound as a new technology is suitable for aiding this process, but its diagnostic value remains unclear. We searched MEDLINE, EMBASE, Cochrane Central Register of Controlled Trials, and the Chinese biomedical literature database to summarize specific applications of airway ultrasound in each step of endotracheal intubation in pediatric patients. Diagnostic accuracy and 95% confidence interval were used as outcomes. In total, 33 studies (6 randomized controlled trials and 27 diagnostic studies) with 1934 airway ultrasound examinations were included. Population included neonates, infants, and older children. Airway ultrasound could be used to determine the endotracheal tube size and confirm endotracheal intubation and intubation depth; the diagnostic accuracy for all these factors was 23.3–100%, 90.6–100%, and 66.7–100%, respectively. Furthermore, the accuracy of airway ultrasound in predicting endotracheal tube size was consistently higher than traditional methods, such as height formula, age formula, and the width of the little finger. In conclusion, airway ultrasound has unique advantages for confirming successful endotracheal intubation in pediatric patients, and it may become an effective auxiliary tool in this field. There is a need to develop a unified airway ultrasound protocol to conduct clinical trials and practice in the future.

## 1. Introduction

Endotracheal intubation is challenging in pediatric patients. Improper ET size may cause airway injury and poor ventilation, which increase the risk of airway stenosis and pulmonary complications. Esophageal intubation and endobronchial intubation may also lead to adverse events, and even fatal threats to children. Previous data show that the incidence of the endotracheal tube (ET) being placed too shallow (above the inferior clavicular border) or too deep (at or below the carina) is as high as 35% in children under one year old [1], and the success rate of first intubation performed by junior residents in emergency pediatric patients and neonates is less than 50% [2,3].

There is no proper method to select appropriate ET size for pediatric patients. Traditional methods, such as the height and age formula, are not accurate enough [4,5]. End-tidal CO_2_ (ETCO_2_) and chest X-ray (CX) are the gold standards for checking endotracheal intubation [6] and determining intubation depth [7,8], respectively. However, the value of ETCO_2_ can be misleading in some cases such as pulmonary embolism, cardiac arrest, and residual gas in the stomach. Radiation exposure of CX and a cumbersome machine restrict its use in emergencies. In addition, neither can be used to determine the correct ET size.

Airway ultrasound is starting to gain attention in assisting endotracheal intubation, especially in neonates and young infants, because the sternum is not ossified and the large thymus keeps the lungs away, which allow a clear view of the mediastinal structures. It can also be used to select ET size and locate tube position before intubation and ventilation to avoid changing the tube and abdominal distension.

Some clinical studies that used airway ultrasound to assist endotracheal intubation in pediatric patients have recently emerged, but the diagnostic value of this technology is still unclear. Therefore, we conducted a systematic review of the existing literature.

## 2. Materials and Methods

The protocol of our study was registered in the International Prospective Register of Systematic Reviews (CRD42021258003) in July 2021 based on the Preferred Reporting Items for Systematic Reviews and Meta-Analyses (PRISMA) 2020 statement [9].

### 2.1. Literature Search

We systematically searched MEDLINE via PubMed, EMBASE, the Cochrane Central Register of Controlled Trials, and the Chinese biomedical literature database for studies dated from inception to October 2021. We did not apply any language restrictions. The search terms were “ultrasonography” and “intratracheal intubation.” A search filter was used to limit the age from zero to eighteen years. The detailed search strategy is given in Supplementary Table S1. We also manually searched the reference lists of retrieved articles.

### 2.2. Eligibility Criteria

Studies were considered eligible if they met the Population, Intervention, Control, Outcome, and Study design (PICOS) criteria [9], which included the following points: (a) Population: pediatric patients (age < 18 years) receiving endotracheal intubation; (b) Intervention: airway ultrasound used to assist endotracheal intubation; (c) Control: gold standard as defined by the study or other methods used to assist endotracheal intubation; (d) Outcome: diagnostic accuracy of airway ultrasound; (e) Study design: diagnostic studies and randomized controlled trials. We only included full-text articles and excluded case reports, reviews, letters, and studies that could have patient duplication from other studies.

### 2.3. Study Selection and Data Extraction

Two researchers (LY and MW) independently reviewed the titles, abstracts, and the full text of articles for eligibility. They also assessed the quality of included articles using the Quality Assessment of Diagnostic Accuracy Studies-2 (QUADAS-2) tool [10]. Any disagreements throughout the screening and quality evaluation processes were discussed with the third researcher and resolved through consensus. For each study, the original data were extracted by one researcher and checked by the other, including publication details (author, year), study design, study aims, clinical setting, sample size and type, intervention details, study findings, and conclusions. As the difficulty and risk of endotracheal intubation vary significantly according to age, we extracted data from neonates, infants (1 month to 1 year), and older children, if possible.

### 2.4. Data Synthesis and Analysis

The data retrieved had a significant clinical and statistical heterogeneity, and most of these studies did not provide data such as sensitivity and specificity. Therefore, we could not perform a meta-analysis and qualitatively represent the systematic review results. Diagnostic accuracy (ACC) of airway ultrasound was calculated from raw proportions and 95% confidence interval (CI) using the Wilson method, reflecting this technology’s clinical value. We estimated odds ratios (ORs) and 95% CI for the studies that confirmed ACC by several approaches. The data were analyzed using Stata (version 14.0) and Review Manager (version 5.3).

## 3. Results

**Figure 1:**
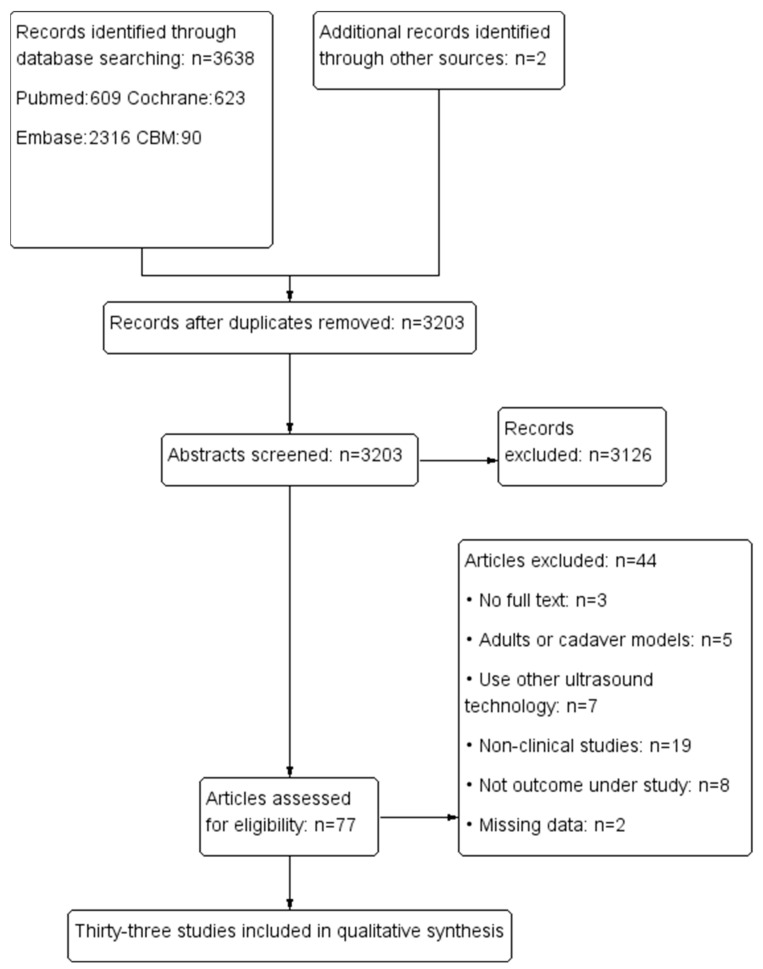
Flow chart of the literature search.

**Figure 2:**
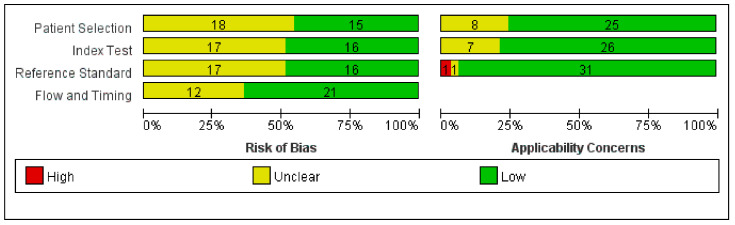
Risk of bias and applicability concerns.

A total of 3638 articles were searched, and 33 articles met our inclusion criteria [4,5,11,12,13,14,15,16,17,18,19,20,21,22,23,24,25,26,27,28,29,30,31,32,33,34,35,36,37,38,39,40,41]. Figure 1 shows a flow chart of the literature search. Six studies were randomized controlled trials (RCTs) [4,11,12,20,25,27] and the others were diagnostic studies [5,13,14,15,16,17,18,19,21,22,23,24,26,28,29,30,31,32,33,34,35,36,37,38,39,40,41], the characteristics of which are summarized in Supplementary Table S2. The risk of bias and applicability concerns are presented in Figure 2 and Supplementary Figure S1. We found that half of the studies had an unclear risk in patient selection, index test, and reference standard, and approximately two-thirds had a low risk of bias in research flow and timing. More than 75% of protocols in the included studies had good clinical applicability in patient selection and index tests. We had concerns about the design of reference standards in one study [29].

### 3.1. Selecting ET Size

We included 6 RCTs [4,11,12,20,25,27] and 16 diagnostic studies [5,13,14,15,16,17,18,19,21,22,23,24,26,28,29,30] that fulfilled our inclusion criteria, involving a total of 1816 patients who underwent airway ultrasound examinations 1503 times. All the studies were conducted in children undergoing elective surgery. Except for four studies that included neonates and infants [14,30], most of the patients in the remaining studies were over 1 year old. The ultrasound inspection method involved placing the linear probe on the anterior midline of the neck and measuring the minimal transverse diameter of the subglottic airway (MTDSA) at the level of the cricoid cartilage. MTDSA was measured at a ventilation pressure of 15 mbar in all studies except one [30], and other studies measured MTDSA with suspended ventilation, resulting in zero airway pressure. Six studies substituted the measured values into a formula obtained from pilot studies to calculate the outer diameter (OD) of the ET [12,14,18,20,24,26]; the remaining sixteen studies directly selected the closest OD of the ET based on the measured value. The air leak test is the gold standard for determining the appropriate ET size, but different studies used various reference values. Singh et al. demonstrated that the accuracy of airway ultrasound in determining the optimal ET size was 100% [5]. In addition, the accuracy in seven studies exceeded 90% [11,12,14,20,21,24,28]. Three studies have compared the accuracy of ultrasound in different age groups and reached a consistent conclusion: there is no difference in the accuracy of ultrasound between children aged 0–1 year and those older than 1 year [15,22,30]. Some studies also validated the accuracy of traditional methods for selecting the appropriate ET size, such as the age and height formulas. The results are displayed as OR and 95% CI in Table 1.

### 3.2. Confirming Endotracheal Intubation

Four articles were included, and one hundred and seventy-one patients underwent airway ultrasound two hundred and eighteen times, of which six cases were of esophageal intubation [31,32,33,34]. These studies were conducted separately in an intensive care unit (ICU), emergency department (ED), and elective surgery. The ultrasound techniques used to determine endotracheal intubation included: the “comet tail sign” and “double parallel lines” on the transverse and longitudinal view in the anterior, middle line of the neck [31,32], saline-inflated cuff at the level of the suprasternal notch [33], and gentle motion of the ET tip on the parasternal view [34]. Different studies employed various gold standards. The accuracy of airway ultrasound was 100% (95% CI: 92.9–100.0%) [31] and 90.6% (95% CI 79.7–95.9%) [34] in both studies that used CX as the gold standard, and the examination time of ultrasound was significantly shorter than CX (Supplementary Table S2). The other two studies compared airway ultrasound to ETCO_2_ and fiberoptic bronchoscopy, with 93.5% (95% CI: 79.3–98.2%) [32] and 98.8% (95% CI: 93.6–99.8%) accuracy [33]. Two studies in neonates showed no statistically significant difference in accuracy between ultrasound and ETCO_2_ (*p* = 0.414) [32], but ultrasound was slightly less accurate than CX (90.6% vs. 100%) [34].

### 3.3. Intubation Depth

Seven articles were included in which one hundred and eighty patients underwent airway ultrasound two hundred and thirteen times [35,36,37,38,39,40,41]. All selected studies used CX as the gold standard. Most studies were conducted in an intensive care unit. As it was difficult to clearly show the ET tip and carina simultaneously, it was necessary to use other anatomical landmarks as a reference to evaluate the intubation depth using airway ultrasound. One of the methods was observing the position of the ET tip [35,36,37] or cuff [38] at the level of the sternum notch and measuring the distance from the ET tip to the aortic arch [39,40] or right pulmonary artery [41] on the parasternal longitudinal view. Different studies had different definitions of suitable intubation depth, as shown in Table 2. The number of ultrasound examinations varied greatly among different studies, ranging from 6 [37] to 60 [38]. The diagnostic accuracy of airway ultrasound ranged from 66.7% to 100% across all studies. Five studies were conducted on airway ultrasound to confirm intubation depth in neonates [35,36,37,39,41]. Excluding two studies with fewer than 10 ultrasound examinations [35,37], the accuracy of airway ultrasound determining intubation depth in neonates ranged from 73.3 to 100% [36,39,41].

## 4. Discussion

In general, airway ultrasound is currently the only technology that can simultaneously select suitable ET size and confirm endotracheal intubation and depth of intubation. Two meta-analyses have confirmed that airway ultrasound has a good diagnostic performance in confirming ET position in adults [42,43], of which the pooled sensitivity and specificity were 0.98 and 0.97. This conclusion, however, cannot be directly extended to pediatric patients. In this review, we summarized the specific applications of airway ultrasound in pediatric patients and calculated the ACC and 95% CI to investigate its clinical value.

Most included studies have shown that airway ultrasound can determine ET size with greater than 80% accuracy. This was also confirmed by other studies because cricoid cartilage diameter had a strong relationship with optimal ET OD [44,45]. Furthermore, airway ultrasound predicting ET size was more accurate than traditional methods used in the same study. Rajasekhar et al. reported the lowest ACC of ultrasound and little finger width [29]. The most likely reason for this result was the lower limit of the air leak test, which was set to 20 cm H_2_O, and there was no upper limit in the study method, which might lead to a larger ET size determined by the gold standard [29].

Airway ultrasound has been recommended as an alternative method for determining whether an ET is in the trachea in two guidelines for adult patients [6,46]. In the absence of a professional sonographer, the diagnostic ACC of airway ultrasound reported in included studies was greater than 90%, which may provide an update for guidelines. There were only six cases of esophageal intubation in all included studies, and the ability of airway ultrasound to detect this potentially fatal error requires further investigation.

It is feasible to use airway ultrasound to determine the intubation depth in neonates. In three studies with the most frequent airway ultrasound examinations, ACC was higher than 90% [38,39,41]. The ultrasound probe placed at the level of the sternal notch can only be used for qualitative evaluation. In contrast, the ultrasound probe placed at the level of the parasternal longitudinal axis can achieve quantitative measurement from ET tip to the carina. Several studies have confirmed a strong relationship between ultrasound and CX measurements [44,47]. However, the quantitative measurement method is not appropriate for older children with sternal ossification. Furthermore, curvilinear and linear probes were used to determine ET position and intubation depth. One study found no statistical difference in the diagnostic accuracy of the two, but the linear probe takes less time [48].

Included studies in this review almost covered all ages of pediatric patients, especially neonates and infants, who are very different from older children. No studies were discontinued due to adverse events, indicating that airway ultrasound is a safe technology. Two studies also confirmed that premature infants can tolerate ultrasound examinations well [49] and airway ultrasound could result in fewer airway-related complications and adverse events [50]. Compared with radiological examination, bedside ultrasound examination allows small adjustments in real time and can reduce the radiation exposure and potential risks associated with critical patient transfer. Additionally, airway ultrasound has a quick diagnostic speed. The average measurement time for MTDSA is 24.9–78.3 s [15,24,27], which can be carried out concurrently with pre-oxygenation. Ultrasound takes significantly less time than CX to determine ET position, which has significant clinical implications in emergencies [31,34]. Other studies have reported that airway ultrasound is even faster than auscultation and ETCO_2_ in determining ET position [51,52]. Finally, during the period of the coronavirus 2019 pandemic, using airway ultrasound in pediatric patients predicted the ET size more accurately and helped avoid unnecessary procedures caused by an inappropriate tube. It could quickly determine the position of the ET before ventilation. This technology can ensure patient safety and reduce the risk of occupational exposure for doctors [53].

It is noteworthy that the three applications of airway ultrasound described above involve different clinical scenarios and study populations. The accuracy of using airway ultrasound to confirm endotracheal intubation, whether in an ICU, ED, or operating room, is satisfactory. Moreover, airway ultrasound can quickly distinguish between esophageal and endotracheal intubation before ventilation. Studies to confirm intubation depth have mainly been conducted in ICUs. Data have shown that the incidence of inappropriate intubation depth in pediatric ICUs is as high as 70% [54]. As a result, it is necessary to rule out changes in respiratory status of neonates due to inappropriate intubation depth. Airway ultrasound can not only detect too deep or shallow intubation, but also reduce radiation exposure, which is suitable for ICUs. Studies using airway ultrasound to select ET size have mainly been conducted in children older than 1 year undergoing elective surgery. Although ultrasound is more accurate in selecting appropriate size than conventional methods, it is of little value to experienced anesthesiologists. However, it may be a good teaching tool for inexperienced interns or residents [55].

In this systematic review, we thoroughly used data from existing studies to add feasibility and effectiveness to the applications of airway ultrasound in endotracheal intubation of pediatric patients. However, there remain some limitations: (1) the outcome of this analysis is in the form of diagnostic accuracy, which may conceal the flaw of insufficient sensitivity or specificity of airway ultrasound. (2) All included studies are single-center studies with small sample size, and half have an unclear risk of bias in the design, which may affect the accuracy of the conclusions. (3) As there is no standard for the use of airway ultrasound, there is a great deal of heterogeneity between studies, including differences in patient’s age, region, and ethnicity, the experience, skill, and training of ultrasound operators, inconsistent gold standards, and various ultrasound examination protocols.

In the future, there is a need to formulate a unified and standardized airway ultrasound examination protocol, diagnostic criteria, and reference tests for pediatric patients. It is essential to investigate the efficacy of airway ultrasound in facilitating endotracheal intubation in pediatric patients via multicenter clinical research with a large sample size. In addition, future studies are needed to explore the population who can benefit from airway ultrasound and clinical scenarios to maximize the value of this technology. Study populations should be stratified according to age, with a focus on neonates and young infants, especially those admitted to the ICU or ED. Limited studies have shown that residents differ significantly in their mastery of the technique [55,56] after receiving brief training in airway ultrasound. Exploring an efficient and standardized training and assessment process is an intriguing direction that will help promote this technology in resident training programs and hospitals.

## 5. Conclusions

In conclusion, the applications of airway ultrasound for endotracheal intubation in pediatric patients are effective and feasible. Future work needs to focus on children of different ages covered in this review. The use of multiple technologies in pediatric endotracheal intubation can ensure patient safety to the greatest extent possible, which is more in line with current clinical practice.

## Figures and Tables

**Table 1:** Diagnostic accuracies of airway ultrasound and traditional methods are shown as odds ratio (OR) and 95% CI.

Traditional Method	Author/Year	n_1_/N_1_^a^	n_2_/N_2_^a^	OR (95% CI) [%]
Cole formula ^b^				
	Chen, 2015 [11]	47/50	27/50	1.38 (0.96–1.99)
	Gnanaprakasam, 2017 [4]	56/75	34/75	1.37 (0.97–1.93)
	Shen, 2015 [12]	49/50	27/50	1.41 (0.98–2.03)
	Singh, 2019 [5]	100/100	95/100	1.03 (0.84–1.25)
	Cho, 2015 [18]	78/126	42/126	1.53 (1.12–2.09)
	Elshazly, 2020 [20]	23/25	17/25	1.18 (0.74–1.90)
	Schramm, 2012 [22]	24/50	12/50	1.68 (0.91–3.07)
	Makireddy, 2020 [23]	29/41	27/41	1.04 (0.70–1.56)
	Raksamani, 2018 [27]	37/47	24/46	1.28 (0.86–1.92)
Penlington’s formula ^c^				
	Zhang YJ, 2017 [25]	35/40	21/40	1.36 (0.89–2.07)
	Schramm, 2012 [22]	24/50	20/50	1.14 (0.69–1.86)
Motoyama formula ^d^				
	Zhang K, 2017 [17]	48/60	33/60	1.25 (0.89–1.77)
Height formula ^e^				
	Sutagatti, 2017 [13]	67/75	27/75	1.78 (1.23–2.57)
	Singh, 2019 [5]	100/100	81/100	1.12 (0.90–1.38)
	Laksono, 2020 [21]	12/13	9/14	1.23 (0.64–2.36)
The width of little finger ^f^				
	Rajasekhar, 2018 [29]	14/60	11/60	1.22 (0.59–2.51)
	Singh, 2019 [5]	100/100	98/100	1.01 (0.83–1.23)
	Laksono, 2020 [21]	12/13	9/13	1.17 (0.61–2.24)

^a^ n_1_/N _1_: Accuracy of airway ultrasound; n_2_/N_2_: Accuracy of traditional methods; ^b^ Cole formula: ET inner diameter (ID) [mm] = 4 + (age[years]/4); ^c^ Penlington’s formula: ET ID [mm] = 4.5 + (age[years]/4); ^d^ Motoyama formula: ET ID [mm] = 3.5 + (age[years]/4); ^e^ Height formula: ET ID [mm] = 2 + (height[cm]/30); ^f^ The width of little finger: selecting the closest ET outer diameter.

**Table 2:** Definitions of suitable intubation depth.

Author/Year	Airway Ultrasound	Chest X-ray
Saul, 2016 [35]	ETT tip below suprasternal notch, above carina	ETT tip ≥ 2 cm below vocal cords, above carina
de Kock, 2015 [36]	ETT tip inferior to thyroid, superior to aortic arch	ETT tip at T1/2 level
Lingle, 1988 [37]	ETT tip below suprasternal notch, above superior margin of aortic arch	
Uya, 2020 [38]	Saline-filled cuff at suprasternal notch level	ETT tip below clavicle, ≥1 cm above carina
Chowdhry, 2015 [39]	Distance from apex of aortic arch to ETT tip ≥ 1 cm	ETT tip at or above the body of T3
Slovis, 1986 [40]	Distance from aortic arch to ETT tip was 1 cm	ETT tip below inferior margin of clavicle, ≥0.5 cm above carina
Dennington, 2012 [41]	Not available	ETT tip below thoracic inlet, above carina

## Data Availability

The data used to support the findings of this study are included within the article.

## Supplementary Materials

The following supporting information can be downloaded at: https://www.mdpi.com/article/10.3390/jcm12041477/s1, Table S1: Search strategy. Table S2: Characteristics of included studies. Figure S1: Risk of bias and applicability concerns for each included article.
