# Supplementary material for: Applications of Airway Ultrasound for Endotracheal Intubation in Pediatric Patients: A Systematic Review
Source: J Clin Med. 2023 Feb 13;12(4):1477. doi: 10.3390/jcm12041477 (PMC9961112; doi:10.3390/jcm12041477)

Supplementary Table S1. Search strategy

Pubmed

#	Searches	Results
1	Ultrasonics[MeSH Terms]	24851
2	Ultrasonography[MeSH Terms]	461511
3	(((Ultraso*[Title/Abstract]) OR (Echotomograph*[Title/Abstract])) OR (Sonograph*[Title/Abstract])) OR (Echograph*[Title/Abstract])	450738
4	#1 or #2 or #3	690312
5	Intubation, intratracheal[MeSH Terms]	40782
6	Intubation[MeSH Terms]	55590
7	(((intubation*[Title/Abstract]) OR (endotracheal tube*[Title/Abstract])) OR (tracheal tube*[Title/Abstract])) OR (intratracheal tube*[Title/Abstract])	60095
8	#5 or #6 or #7	89416
9	#4 and #8	2194
10	#4 and #8 and (allchild [Filter])	609

EMBASE

#	Searches	Results
1	'ultrasound'/exp	201995
2	'echography'/exp	911707
3	'echograph'/exp	48252
4	ultraso*:ab,ti OR sonograph*:ab,ti OR echotomograph*:ab,ti OR echograph*:ab,ti	647701
5	'ultrasound'/exp OR 'echography'/exp OR 'echograph'/exp OR (ultraso*:ab,ti OR sonograph*:ab,ti OR echotomograph*:ab,ti OR echograph*:ab,ti)	1250749
6	'respiratory tract intubation'/exp	63463
7	intubation*:ab,ti OR 'endotracheal tube*':ab,ti OR 'tracheal tube*':ab,ti OR 'intratracheal tube*':ab,ti	90110
8	'endotracheal tube'/exp	20792
9	'respiratory tract intubation'/exp OR (intubation*:ab,ti OR 'endotracheal tube*':ab,ti OR 'tracheal tube*':ab,ti OR 'intratracheal tube*':ab,ti) OR 'endotracheal tube'/exp	126306
10	('ultrasound'/exp OR 'echography'/exp OR 'echograph'/exp OR (ultraso*:ab,ti OR sonograph*:ab,ti OR echotomograph*:ab,ti OR echograph*:ab,ti)) AND ('respiratory tract intubation'/exp OR (intubation*:ab,ti OR 'endotracheal tube*':ab,ti OR 'tracheal tube*':ab,ti OR 'intratracheal tube*':ab,ti) OR 'endotracheal tube'/exp)	9266
11	('ultrasound'/exp OR 'echography'/exp OR 'echograph'/exp OR (ultraso*:ab,ti OR sonograph*:ab,ti OR echotomograph*:ab,ti OR echograph*:ab,ti)) AND ('respiratory tract intubation'/exp OR (intubation*:ab,ti OR 'endotracheal tube*':ab,ti OR 'tracheal tube*':ab,ti OR 'intratracheal tube*':ab,ti) OR 'endotracheal tube'/exp) AND ([newborn]/lim OR [infant]/lim OR [child]/lim OR [adolescent]/lim)	2316

Cochrane Central Register of Controlled Trials

#	Searches	Results
1	MeSH descriptor: [Ultrasonography] explode all trees	14245
2	MeSH descriptor: [Ultrasonics] explode all trees	314
3	(Ultraso*):ti,ab,kw OR (Echotomograph*):ti,ab,kw OR (Sonograph*):ti,ab,kw OR (Echograph*):ti,ab,kw	48318
4	#1 or #2 or #3	52613

5	MeSH descriptor: [Intubation, Intratracheal] explode all trees	4568
6	MeSH descriptor: [Intubation] explode all trees	5426
7	(intubation*):ti,ab,kw OR (endotracheal tube*):ti,ab,kw OR (tracheal tube*):ti,ab,kw OR (intratracheal tube*):ti,ab,kw	19852
8	#5 or #6 or #7	20354
9	#4 and #8 in Trials	623

Chinese biomedical literature database

#	Searches	Results
1	″Intubation, Intratracheal″	20055
2	″endotracheal intubation″OR ″endotracheal tube″ OR ″intubation depth″ OR ″position″ OR ″tube diameter″ OR ″tube size″ OR ″tube type″	136678
3	(#2) OR (#1)	141239
4	″ Ultrasonics″ OR ″ Ultrasonography″	82032
5	″ultrasound″ OR ″point of care ultrasound″ OR ″airway ultrasound″	371335
6	(#5) OR (#4)	371942
7	(#6) AND (#3)	11468
8	((#6) AND (#3)) AND (clinical trial[article type]) AND (″newborn″[Filter] OR″ child″[Filter] OR ″infant″[Filter] OR "preschool child"[Filter] OR ″adolescent″[Filter]) AND (human[Filter])	90

Supplementary Table S2. Characteristics of included studies

Study purpose	Author/ Year	Study type	Airway US exams	Age [a]	Usage scenario	Ultrasound protocol/Probe type	Operators	Gold standard	Accuracy (95%CI) [%]	Other outcomes
ET size										
	Chen/ 2015[11]	RCT	50	1.6±1.7y	Elective operation	Measure MTDSA/Linear, 12MHz	Sonographer	Air leak test 10-20cmH2O	94.0(83.8-97.9)	Intubation-related complications: US 14% vs Age formula 16%（P ＞0.05）
	Gnanaprakasam/2017[4]	RCT	75	3.5±1.1y	Elective operation	Measure MTDSA/Linear, 8-15MHz	Senior anesthesiologist	Air leak test 10-20cmH2O	74.7(63.8-83.1)	NA
	Shen/ 2015[12]	RCT	50	1.5±1.6y	Elective operation	Measure MTDSA and substitute into formula/Linear, 12MHz	Sonographer	Air leak test 10-20cmH2O	98.0(89.5-99.6)	Intubation-related complications: US 14% vs Age formula 14%（P ＞0.05）
	Sutagatti/ 2017[13]	Diagnostic study	75	10.2±3.2y	Elective operation	Measure MTDSA/Linear, 5-12MHz or 7-15 MHz	Experienced radiologist	Air leak test 10-20cmH2O	89.3(80.3-94.5)	NA
	Shibasaki/2010[14]	Diagnostic study	96	1m-6y	Elective operation	Measure MTDSA and substitute into formula/Linear, 5-10MHz	Sonographer	Air leak test 10-20cmH2O	96.9(91.2-98.9)	NA
	Pillai/2018[15]	Diagnostic study	49	19.4±16.8m	Elective operation	Measure MTDSA/Linear, 8-18MHz	Anesthesiologist with airway US experience	Air leak test 10-20cmH2O	87.8(75.8-94.3)	Mean time from using airway US to selecting ET size is 43s
	Singh/2019[5]	Diagnostic study	100	26.9±16.2m	Elective operation	Measure MTDSA/Linear, 7-15MHz	Experienced radiologist	Air leak test 10-20cmH2O	100.0(96.3-100.0)	NA
	Zhang/2019[16]	Diagnostic study	60	2.4±1.6y	Elective operation	Measure MTDSA/Linear, 8-13MHz	Anesthesiologist with airway US experience	Air leak test 10-20cmH2O	78.3(66.4-86.9)	NA
	Zhang K/2017[17]	Diagnostic study	60	2.3±1.5y	Elective operation	Measure MTDSA/Linear, 13MHz	NA	Air leak test 10-20cmH2O	80.0(68.2-88.2)	NA
	Cho/2015[18]	Diagnostic study	126	79.8±27.1m	Elective operation	Measure MTDSA and substitute into formula/Linear, 5-13MHz	US-trained anesthesiologist	Air leak test 10-25cmH2O	61.9(53.2-69.9)	NA
	Altun/2017[19]	Diagnostic study	152	5.5±2.3y	Elective operation	Measure MTDSA/Linear, 12MHz	Anesthesiologist with airway US experience	Air leak test 10-25cmH2O	88.2(82.1-92.4)	NA
	Elshazly/2020[20]	RCT	25	2.1±1.6y	Elective operation	Measure MTDSA and substitute into formula/Linear, 10-13MHz	Anesthesiologist with airway US experience	Air leak test 10-30cmH2O	92.0(75.0-97.8)	Time of intubation: US 2.5±0.5min vs Age formula 2.2±0.2min（P=0.004）
	Laksono/2020[21]	Diagnostic study	13	2.1±1.6y	Elective operation	Measure MTDSA/Linear, 4.5-13MHz	NA	Air leak test 10-30cmH2O	92.3(66.7-98.6)	NA

	Schramm/2012[22]	Diagnostic study	50	1.5±1.6y	Elective operation	Measure MTDSA/Linear, 6-13MHz	One researcher	Air leak test 15-25 cmH_2O	48.0(34.8-61.5)	NA
	Makireddy/2020[23]	Diagnostic study	41	3.1±1.3y	Elective operation	Measure MTDSA/Linear, 5-13MHz	One researcher	Air leak test 15-25cmH_2O	70.7(55.5-82.4)	NA
	Altun/2021[24]	Diagnostic study	73	5.7±2.0y	Elective operation	Measure MTDSA and substitute into formula/Linear, 5-18MHz or 4-12MHz	Senior anesthesiologist	Air leak test 15-25cmH_2O	94.5(86.7-97.8)	Mean time of measuring MTDSA by US: 24.9±4.6s
	Zhang YJ/2017[25]	RCT	40	4.1±1.5y	Elective operation	Measure MTDSA/Linear, 5-12MHz	Sonographer	Air leak test 15-25cmH_2O	87.5(73.9-94.5)	Intubation-related complications: US 9% vs Age formula 20%（$P > 0.05$）
	Bae/2011[26]	Diagnostic study	100	44.0±31.0m	Elective operation	Measure MTDSA and substitute into formula/Linear, 8-13MHz	NA	Air leak test 15-30cmH_2O	60.0(50.2-69.1)	NA
	Raksamani/2018[27]	RCT	47	2.5±1.4y	Elective operation	Measure MTDSA/Linear, 6-13MHz	US-trained anesthesiologist	Air leak test 20-25cmH_2O	78.7(65.1-88.0)	Mean time of measuring MTDSA by US: 78.3±31.9s
	Gollu/2018[28]	Diagnostic study	61	12.0±4.2y	Elective operation	Measure MTDSA/Linear, 5-13MHz	US-trained anesthesiologist	Air leak test 20-30cmH_2O	98.4(91.3-99.7)	NA
	Rajasekhar/2018[29]	Diagnostic study	60	54.7±47 m	Elective operation	Measure MTDSA/Linear, 7-15MHz	Experienced radiologist	Air leak test 20cmH_2O	23.3(14.4-35.4)	NA
	Schramm/2017[30]	Diagnostic trial	100	21d-6y	Elective operation	Measure MTDSA/Linear, 3-12MHz	Researcher	Air leak test (cutoff value is NA)	83.0(74.5-89.1)	NA
ET position										
	Galicinao/2007[31]	Diagnostic study	50	3.8±5.1y	ED	Comet head and tail sign and double parallel lines /Curvilinear, 5-8 MHz	US-trained and experienced researcher	Chest X-ray	100.0(92.9-100.0)	Mean time of determining ET position: US 17.1s vs X-ray 14.0min
	Alonso/2014[32]	Diagnostic study	31	GA 24-40w in NICU, 3-14y in PICU	ICU	Comet tail sign and double parallel lines/Curvilinear, 8 MHz or linear 12MHz	Pediatrician with US experience	$ETCO_2$	93.5(79.3-98.2)	Mean time of determining ET position: US 12s vs $ETCO_2$ 6s ($P < 0.001$)
	Tessaro/2015[33]	Diagnostic study	84	9m-18y	Elective operation	Saline-inflated cuff at the level of suprasternal notch/Linear, 5-10MHz	Emergency doctor with US experience	Fiberoptic bronchoscopy	98.8(93.6-99.8)	Mean time of US: 4.0s
	Sethi/2014[34]	Diagnostic study	53	3.9±5.5d	ICU	Gentle motion of ET tip/NA	US-trained researcher	Chest X-ray	90.6(79.7-95.9)	Mean time of determining ET position: US 19.3min vs X-ray 47.3min
Intubation depth										
	Saul/2016[35]	Diagnostic study	9	0-26d	ICU	ETT tip below suprasternal notch and above	A fourth-year radiology	Chest X-ray	100.0(70.1-100.0)	Mean time of US: 7min

					carina/Linear, 5-12MHz	resident or an attending radiologist			
de Kock/2015[36]	Diagnostic study	30	2-38d	ICU	ETT tip inferior to thyroid and superior to aortic arch/Curvilinear, 6MHz	Sonographer	Chest X-ray	73.3(55.6-85.8)	NA
Lingle/ 1988[37]	Diagnostic study	6	GA 29-40w	ICU	ETT tip below suprasternal notch and above superior margin of aortic arch/Linear, 5MHz	NA	Chest X-ray	66.7(30.0-90.3)	NA
Uya/2020[38]	Diagnostic study	60	1.5-13y	Elective operation	Saline-filled cuff at suprasternal notch level/Linear, 6-13MHz	Sonographer	Chest X-ray	95.0(86.3-98.3)	NA
Chowdhry/2015[39]	Diagnostic study	56	GA 28.3±4.7w	ICU	Distance from the apex of aortic arch to ETT tip/Curvilinear, 5-8MHz	Sonographer	Chest X-ray	94.6(85.4-98.2)	NA
Slovis/ 1986[40]	Diagnostic study	21	NA	NA	Distance from aortic arch to ETT tip/ linear, 5 or 7.5MHz	NA	Chest X-ray	85.7(65.4-95.0)	Good correlation between the distance measured by US and X-ray (r=0.80, P<0.001)
Dennington/ 2012[41]	Diagnostic study	31	GA 30.2±4.9w	ICU	Distance from superior portion of right pulmonary artery to ETT tip/ Linear, 13MHz	Two US-trained researchers	Chest X-ray	100.0(89.0-100.0)	Linear correlation between the distance measured by US and X-ray (r^2=0.68); Mean time of US lees than 5min

[a] Age is expressed by Mean ± SD or Range.

ED: emergency department; ET: endotracheal tube; ICU: intensive care unit; MTDSA: minimal transverse diameter of the subglottic airway; NA: not available;

RCT: randomized controlled trial; US: ultrasoun

Supplementary Figure S1. Risk of bias and applicability concerns for each included article [4,5,11–41].

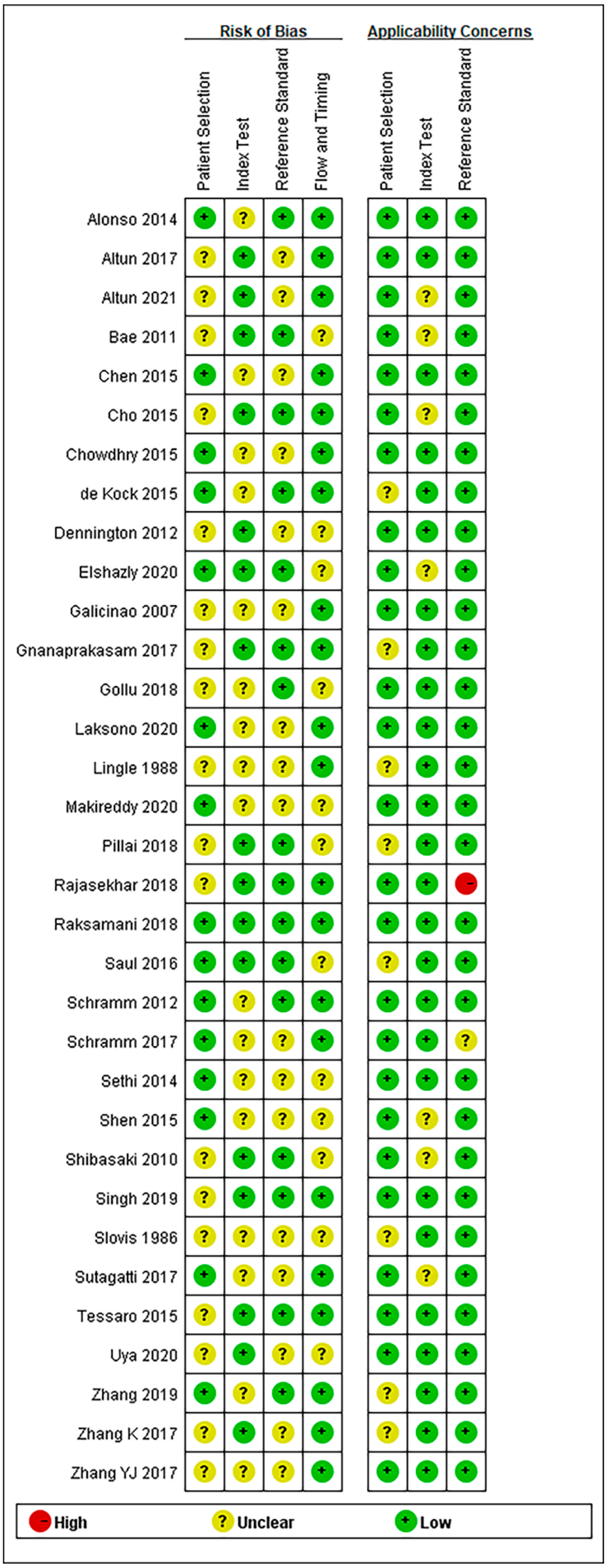

Supplement: Supplementary file 1 [file jcm-12-01477-s001.zip › jcm-2176005-supplementary.pdf]
